# Supplementary material for: The ubiquitin ligase HUWE1 enhances WNT signaling by antagonizing destruction complex-mediated β-catenin degradation and through a mechanism independent of β-catenin stability
Source: bioRxiv. 2024 Mar 17:2024.02.02.578552. Originally published 2024 Feb 2. Preprint. [Version 2] doi: 10.1101/2024.02.02.578552 (PMC10896346; doi:10.1101/2024.02.02.578552)

bioRxiv preprint doi: https://doi.org/10.1101/2024.02.02.578552; this version posted March 17, 2024. The copyright holder for this preprint (which was not certified by peer review) is the author/funder. This article is a US Government work. It is not subject to copyright under 17 USC 105 and is also made available for use under a CC0 license.

S1 Fig. HUWE1 and AXIN1 reciprocally regulate WNT signaling by modulating GSK3A/GSK3B-dependent CTNNB1 phosphorylation and abundance.

bioRxiv preprint doi: https://doi.org/10.1101/2024.02.02.578552; this version posted March 17, 2024. The copyright holder for this preprint (which was not certified by peer review) is the author/funder. This article is a US Government work. It is not subject to copyright under 17 USC 105 and is also made available for use under a CC0 license.

(A-C) We note that the data for WT HAP-7TGP, CSNK1A1^{KO} and CSNK1A1^{KO}; HUWE1^{KO} cells is discussed in the first section of the results, while the data for CSNK1A1^{KO}; AXIN1^{OE} and CSNK1A1^{KO}; HUWE1^{KO}; AXIN1^{OE} cells is discussed in a later section of the results subtitled "HUWE1 enhances WNT signaling by antagonizing the DC." Cells were treated with DMSO vehicle or 10 μM of the GSK3A/GSK3B inhibitor CHIR-99021 for 48 hr as indicated. (A) Immunoblots of soluble CTNNB1 from MFS, used for quantification in Fig 1B. (B) Immunoblots of non-phospho-CTNNB1 (S33/S37/T41) and total CTNNB1 from WCE, used for quantification in Fig 1C and S1C Fig, respectively. (C) Total CTNNB1 abundance (CTNNB1 intensity normalized to total protein, average ± SD from duplicate immunoblots shown in S1B) in WCE of the indicated cell lines, relative to WT HAP1-7TGP cells treated with DMSO. (D) Immunoblot analysis of total AXIN1 from WCE of the indicated cell lines used in A-C, and in Fig 1. The polyclonal cell populations overexpressing AXIN1 were generated as described in Materials and methods. AXIN1 abundance (AXIN1 intensity normalized to GAPDH intensity), relative to CSNK1A1^{KO} cells, is indicated below the blots.

bioRxiv preprint doi: https://doi.org/10.1101/2024.02.02.578552; this version posted March 17, 2024. The copyright holder for this preprint (which was not certified by peer review) is the author/funder. This article is a US Government work. It is not subject to copyright under 17 USC 105 and is also made available for use under a CC0 license.

S2 Fig. HUWE1 enhances WNT signaling through a mechanism independent of CTNNB1 stability.

(A) Genomic nucleotide and corresponding amino acid sequences comprising the CTNNB1 phosphodegron of WT HAP1-7TGP and CTNNB1$^{ST\text{-}A}$ cells. The kinases that phosphorylate S or T residues in the phosphodegron are indicated. Nucleotides and amino acids in red indicate mutations. (B) Immunoblots of soluble HUWE1 and CTNNB1 from MFS of the indicated cell lines. The CTNNB1 immunoblots were used for quantification in Fig 2A. (C-E) Treatment of CTNNB1$^{ST\text{-}A}$ cells with WNT3A does not promote further accumulation of soluble CTNNB1 and does not further increase WNT target gene expression. Cells were treated with 50% WNT3A CM for 24 hr where indicated. (C) Immunoblots of total CTNNB1 from WCE used for quantification in D. (D) Total CTNNB1 abundance (CTNNB1 intensity normalized to total protein and GAPDH intensity, average ± SD from duplicate lanes of the immunoblots shown in C) in WCE of CTNNB1$^{ST\text{-}A}$ cells treated with WNT3A CM, relative to untreated CTNNB1$^{ST\text{-}A}$ cells. Significance was determined by unpaired t-test with Welch's correction. (E) mRNA abundance (average ± SD *AXIN2*, *RNF43*, *TNFRSF19*, or *NKD1* mRNA normalized to *HPRT1* mRNA, each measured in triplicate reactions) in CTNNB1$^{ST\text{-}A}$ cells treated with WNT3A CM,

bioRxiv preprint doi: https://doi.org/10.1101/2024.02.02.578552; this version posted March 17, 2024. The copyright holder for this preprint (which was not certified by peer review) is the author/funder. This article is a US Government work. It is not subject to copyright under 17 USC 105 and is also made available for use under a CC0 license.

reported as percentage of the mRNA abundance in untreated CTNNB1^{ST-A} cells. (F) WNT reporter activity (median EGFP fluorescence from 5,000 singlets) for the indicated cell lines, relative to the average for CTNNB1^{ST-A} cells. Each circle represents a unique clonal cell line (determined by genotyping, S1 File), and the average of 9-12 independent clones for each genotype is indicated by a horizontal line and quantified above each group of circles. Significance was determined by unpaired t-test with Welch's correction.

bioRxiv preprint doi: https://doi.org/10.1101/2024.02.02.578552; this version posted March 17, 2024. The copyright holder for this preprint (which was not certified by peer review) is the author/funder. This article is a US Government work. It is not subject to copyright under 17 USC 105 and is also made available for use under a CC0 license.

S4 Fig. HUWE1 enhances WNT signaling through mechanisms mediated by a subset of DC components including APC, AXIN1 and GSK3A or GSK3B.

(A) Immunoblot analysis of total protein from WCE of the indicated clonal cell lines used for CRISPRi-mediated HUWE1 KD in Fig 4 and S5 Fig. The AXIN1 and AXIN2 immunoblots of CSNK1A1^{KO}; AXIN1^{KO} and CSNK1A1^{KO}; AXIN2^{KO} cells, respectively, exhibited bands of lower abundance and molecular weight than their respective counterparts in WT HAP1-7TGP cells. These bands may represent residual truncated protein products, but in both cases frameshift mutations in the single allele of the respective genes (S1 File) predicted the absence of full-

bioRxiv preprint doi: https://doi.org/10.1101/2024.02.02.578552; this version posted March 17, 2024. The copyright holder for this preprint (which was not certified by peer review) is the author/funder. This article is a US Government work. It is not subject to copyright under 17 USC 105 and is also made available for use under a CC0 license.

length, WT proteins. * indicates a non-specific band observed with the rabbit anti-APC antibody. The "a" and "b" superscripts next to the protein names indicate which of two membranes the corresponding strips were cut from. Dashed vertical lines indicate a rearrangement of samples within the same blot. (B, C) GSK3A and GSK3B are functionally redundant in WNT signaling in HAP1 cells. The same cell lines were used in B and C. (B) Immunoblot analysis of total GSK3A and GSK3B from WCE of the indicated cell lines. (C) WNT reporter activity (median EGFP fluorescence from 50,000 singlets was measured for biological duplicates of a single clone, and the average ± SD of the two measurements was calculated) relative to untreated WT HAP1-7TGP cells. (D) HUWE1 abundance, quantified by dot blots, in the clonal cell lines used for CRISPRi-mediated HUWE1 KD in Fig 4 and S5 Fig. Total HUWE1 abundance (HUWE1 intensity normalized to total protein, average ± SD from triplicate dot blots) in WCE of the indicated cell lines, relative to WT HAP1-7TGP cells. Significance was determined by unpaired t-test with Welch's correction, and the difference in HUWE1 abundance between each mutant cell line and WT HAP1-7TGP cells was not significant (not depicted).

S5 Fig. Quantification of CRISPRi-mediated HUWE1 KD in various genetic backgrounds. (A-B) Two polyclonal cell populations targeted with HUWE1 sgRNAs (1 and 2) and one polyclonal cell population targeted with SCR sgRNA were derived for each genotype as described in Materials and methods. (A) Immunoblots of total HUWE1 from WCE used for quantification in B. The "a" and "b" superscripts next to the protein names indicate which of two duplicate membranes the corresponding strips were cut from. Dashed vertical lines indicate a rearrangement of samples within the same blot. (B) HUWE1 abundance (average HUWE1 intensity normalized to either Na^+/K^+ ATPase or GAPDH intensity from duplicate immunoblots

bioRxiv preprint doi: https://doi.org/10.1101/2024.02.02.578552; this version posted March 17, 2024. The copyright holder for this preprint (which was not certified by peer review) is the author/funder. This article is a US Government work. It is not subject to copyright under 17 USC 105 and is also made available for use under a CC0 license.

shown in A) in WCE of cell populations targeted with HUWE1 sgRNAs, reported as percentage of HUWE1 abundance in WCE of cell populations targeted with SCR sgRNA control.

S6 Fig. Quantification of CRISPR/Cas9-mediated *HUWE1* mutations in HEK293T-7TG and HEK293T-7TG CSNK1A1^{KO} cells.

(A, B) Sequencing reads of the *HUWE1* locus targeted by CRISPR/Cas9 in individual clonal cell lines derived from HEK293T-7TG (A) or HEK293T-7TG CSNK1A1^{KO} (B) cells were quantified for mutations. The X-axis shows individual clones, and the Y-axis indicates the percentage of reads containing mutations. Bars in dark blue indicate the percentage of reads containing any kind of mutation (total mutations) at the targeted locus in each clone, and bars in light blue indicate the percentage of reads containing out-of-frame mutations at the same locus. In all 113 clones in which ~100% of the reads contained mutations (indicating all *HUWE1* alleles had been successfully targeted), some of those mutations were always in frame, strongly suggesting that at least one WT *HUWE1* allele is required for cell viability in HEK293T cells.

bioRxiv preprint doi: https://doi.org/10.1101/2024.02.02.578552; this version posted March 17, 2024. The copyright holder for this preprint (which was not certified by peer review) is the author/funder. This article is a US Government work. It is not subject to copyright under 17 USC 105 and is also made available for use under a CC0 license.

Supporting Information:

S1 File. CRISPR/Cas9-engineered clonal cell lines used in this study.

Single-mutant clones in which a single gene was targeted using CRISPR/Cas9 and double- or triple-mutant clones in which multiple genes were targeted using CRISPR/Cas9 are described in two separate spreadsheets labeled accordingly. When more than one clone was generated using the same CRISPR guide, the 'Clone Name' column indicates the generic name used throughout the manuscript to describe the genotype, and the 'Clone #' column identifies an individual clone. The 'HDR Donor' column indicates the name of the ssODN donor template used to generate some of the clonal cell lines (see Materials and Methods). The 'CRISPR guide' column indicates the name of the guide used, which is the same as that of the oligos encoding sgRNAs (see Materials and methods, and S2 File). The 'Genomic Sequence' column shows 80 bases of genomic sequence (5' relative to the gene is to the left) surrounding the target site. For each group of clones made using the same CRISPR guide (separated by gray spacers), the 'Genomic Sequence' column is headlined by the reference WT genomic sequence (obtained from RefSeq), with the guide sequence colored blue. The site of the double strand cut made by Cas9 is between the two underlined bases. Sequencing results for individual clones are indicated below the reference sequence. Some clones that remained WT at the targeted locus are indicated as such and were used as controls. For mutant clones, mutated bases are colored red (dashes represent deleted bases, three dots are used to indicate that a deletion continues beyond the 80 bases of sequence shown, and large insertions are indicated in brackets), and the nature of the mutation and the resulting genotype are described in the columns labeled accordingly. The figures in which each clone was used are also indicated. For double- and triple-mutant clones, the CRISPR

bioRxiv preprint doi: https://doi.org/10.1101/2024.02.02.578552; this version posted March 17, 2024. The copyright holder for this preprint (which was not certified by peer review) is the author/funder. This article is a US Government work. It is not subject to copyright under 17 USC 105 and is also made available for use under a CC0 license.

guide used, the genomic sequence, the mutation and the genotype pertaining to each of the two or three targeted loci are designated '1', '2' and '3' in the column headings, and are shown under green, orange and purple spacers, respectively.

S2 File. Oligonucleotides and primers used in this study.

Oligonucleotides and primers used for generation and characterization of clonal cell lines engineered using CRISPR/Cas9 nuclease (CRISPRn), base editing, and CRISPRi, as well as those used for qRT-PCR, are described in separate spreadsheets labeled accordingly. CRISPRn, base editing and CRISPRi: the names and sequences of pairs of oligonucleotides encoding sgRNAs, which were cloned into the respective vectors for each application as described in Materials and methods, are shown in columns A and B, respectively. Additionally, for CRISPRn and base editing the names and sequences of pairs of forward and reverse primers used to amplify corresponding genomic regions flanking sgRNA target sites are shown in columns C and D, respectively, and where applicable, the names and sequences of individual primers used to sequence the amplified target sites are shown in columns E and F, respectively. qRT-PCR: the names and sequences of pairs of forward and reverse primers used for qRT-PCR are shown in columns A and B, respectively.

S1 Fig

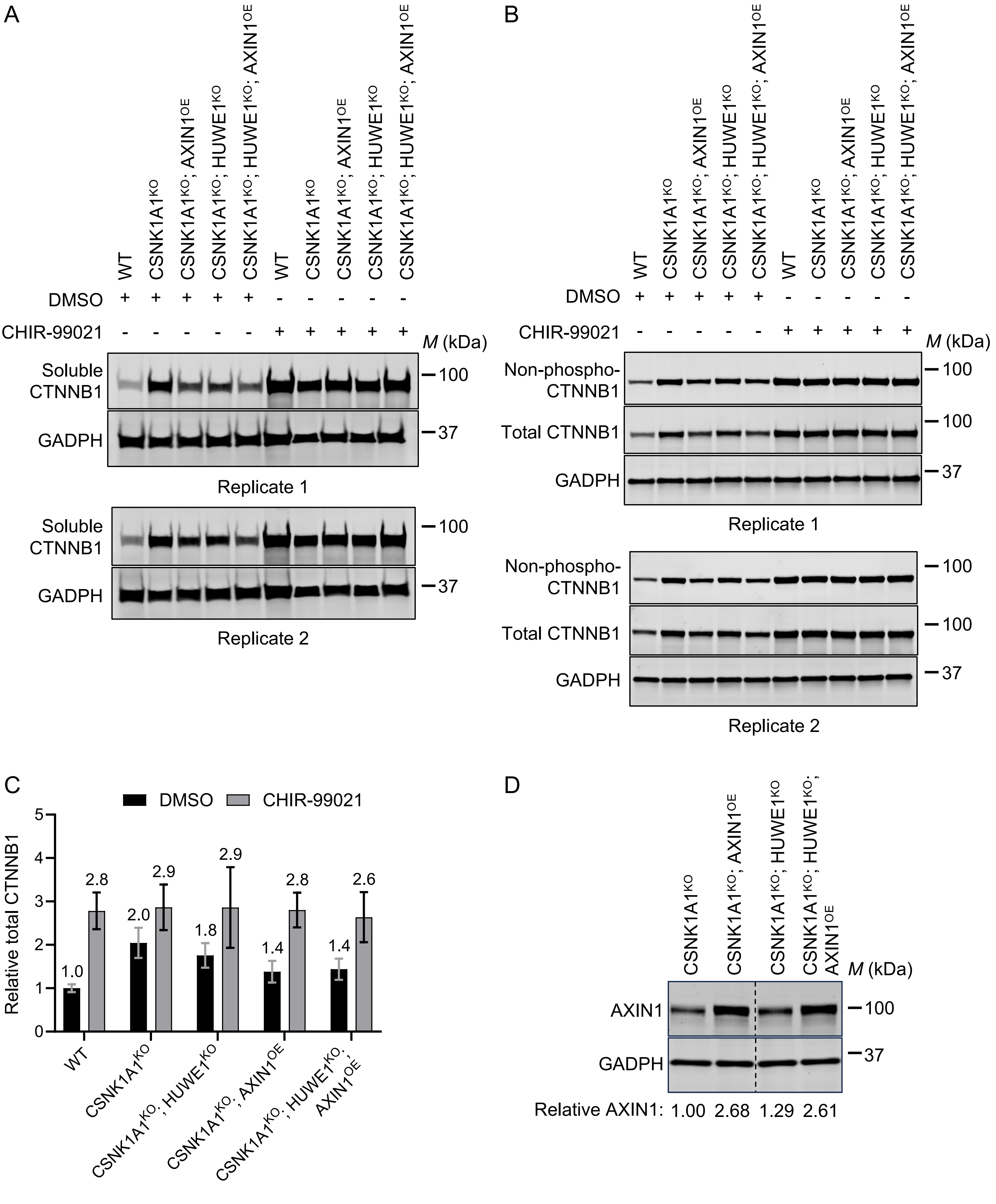
A
WT
CSNK1A1KO
CSNK1A1KO; AXIN1OE
CSNK1A1KO; HUWE1KO
CSNK1A1KO; HUWE1KO; AXIN1OE
DMSO
CHIR-99021
M (kDa)
Soluble CTNNB1
GADPH
100
37
Replicate 1
Replicate 2
B
Non-phospho-CTNNB1
Total CTNNB1
GADPH
Replicate 1
Replicate 2
C
DMSO
CHIR-99021
Relative total CTNNB1
1.0
2.8
2.0
2.9
1.8
2.9
1.4
2.8
1.4
2.6
WT
CSNK1A1KO
CSNK1A1KO; HUWE1KO
CSNK1A1KO; AXIN1OE
CSNK1A1KO; HUWE1KO; AXIN1OE
D
AXIN1
GADPH
Relative AXIN1: 1.00 2.68 1.29 2.61

S2 Fig

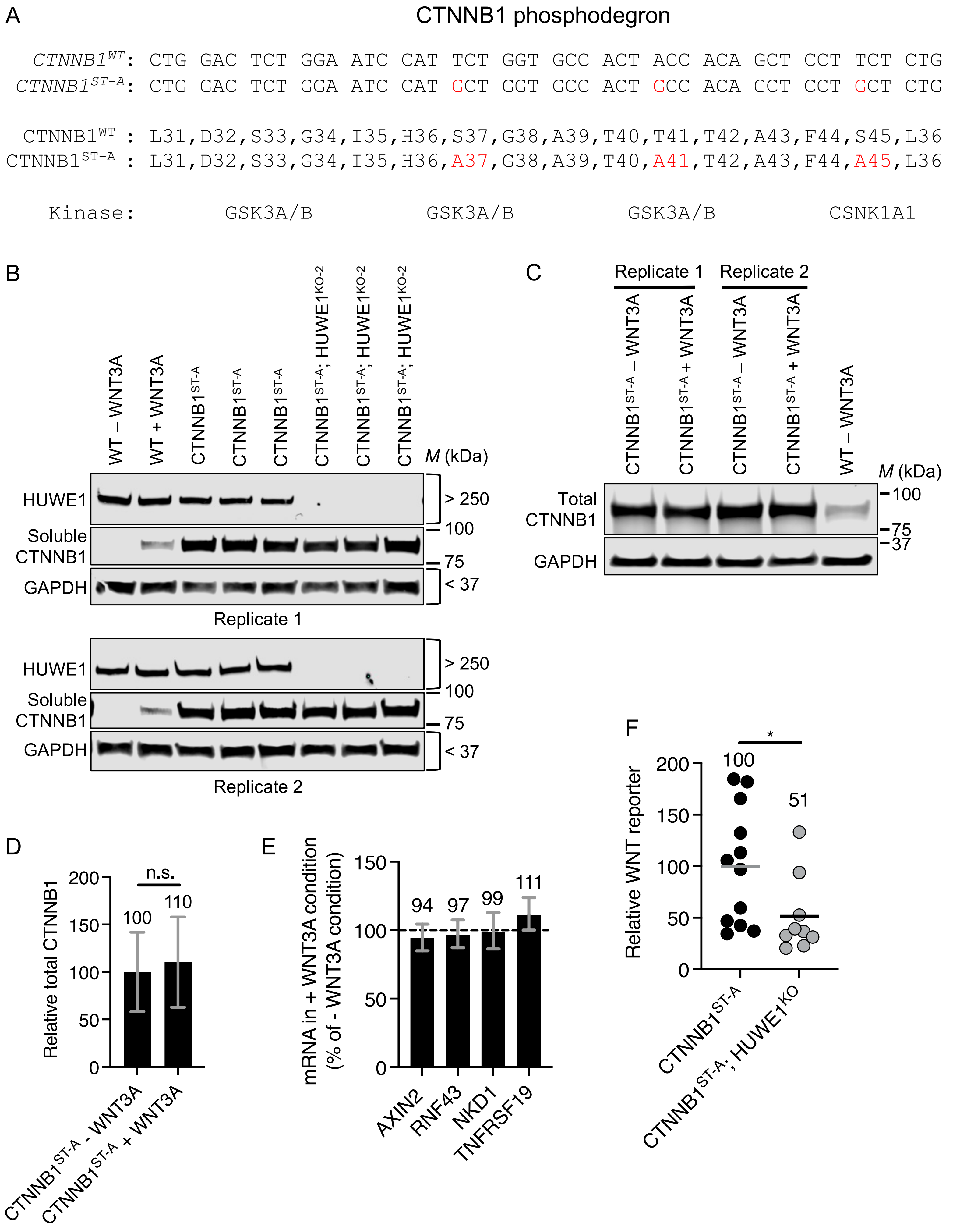
A
CTNNB1 phosphodegron
CTNNB1WT: CTG GAC TCT GGA ATC CAT TCT GGT GCC ACT ACC ACA GCT CCT TCT CTG
CTNNB1ST-A: CTG GAC TCT GGA ATC CAT GCT GGT GCC ACT GCC ACA GCT CCT GCT CTG
CTNNB1WT : L31,D32,S33,G34,I35,H36,S37,G38,A39,T40,T41,T42,A43,F44,S45,L36
CTNNB1ST-A : L31,D32,S33,G34,I35,H36,A37,G38,A39,T40,A41,T42,A43,F44,A45,L36
Kinase: GSK3A/B GSK3A/B GSK3A/B CSNK1A1
B
WT – WNT3A
WT + WNT3A
CTNNB1ST-A
CTNNB1ST-A
CTNNB1ST-A
CTNNB1ST-A; HUWE1KO-2
CTNNB1ST-A; HUWE1KO-2
CTNNB1ST-A; HUWE1KO-2
M (kDa)
HUWE1
Soluble CTNNB1
GAPDH
> 250
100
75
< 37
Replicate 1
HUWE1
Soluble CTNNB1
GAPDH
> 250
100
75
< 37
Replicate 2
C
Replicate 1
Replicate 2
CTNNB1ST-A – WNT3A
CTNNB1ST-A + WNT3A
CTNNB1ST-A – WNT3A
CTNNB1ST-A + WNT3A
WT – WNT3A
M (kDa)
Total CTNNB1
GAPDH
100
75
37
D
Relative total CTNNB1
n.s.
100
110
0
50
100
150
200
CTNNB1ST-A - WNT3A
CTNNB1ST-A + WNT3A
E
mRNA in + WNT3A condition (% of - WNT3A condition)
0
50
100
150
94
97
99
111
AXIN2
RNF43
NKD1
TNFRSF19
F
Relative WNT reporter
*
100
51
0
50
100
150
200
CTNNB1ST-A
CTNNB1ST-A; HUWE1KO

S4 Fig

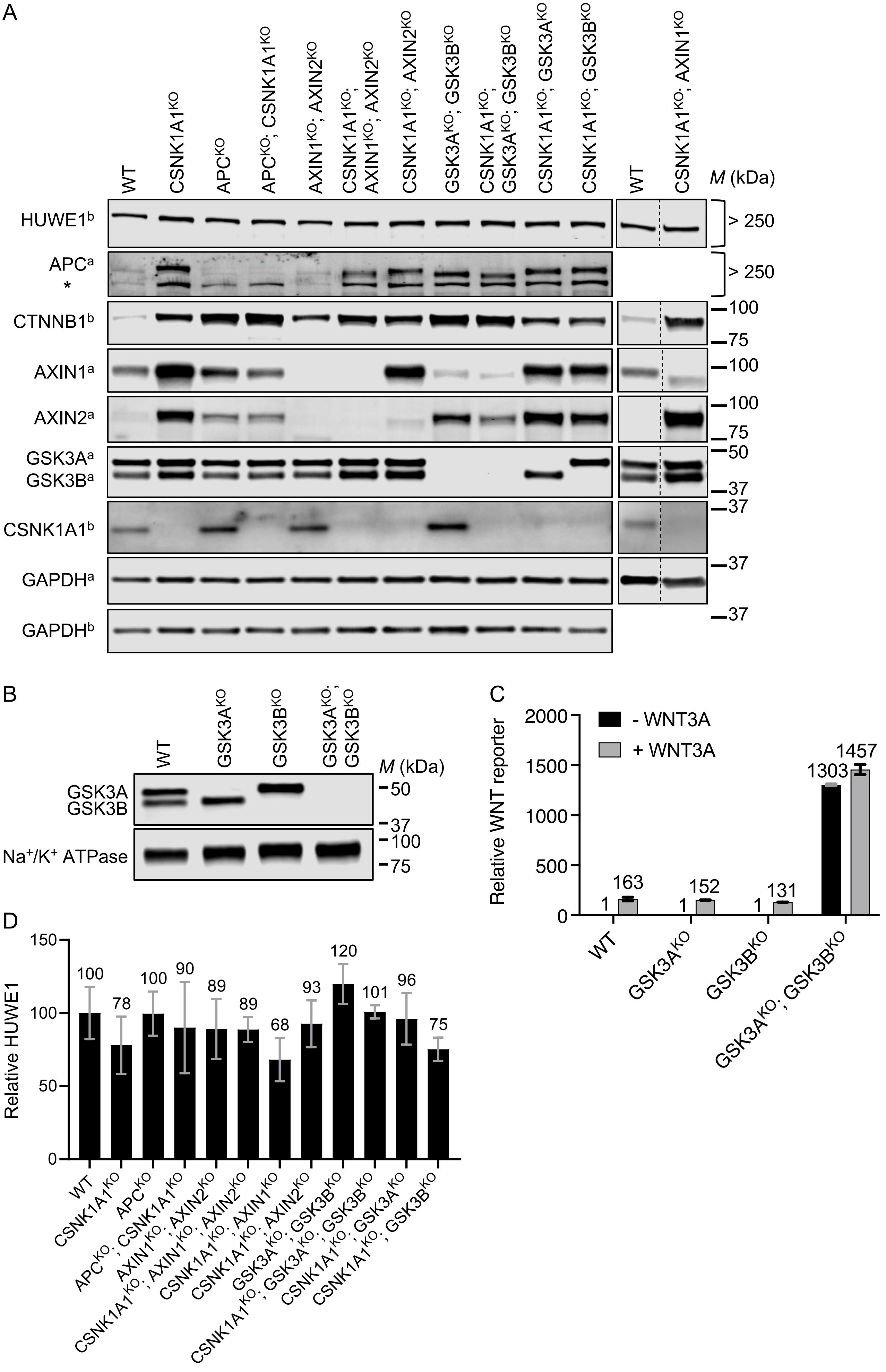
A
WT
CSNK1A1KO
APCKO
APCKO; CSNK1A1KO
AXIN1KO; AXIN2KO
CSNK1A1KO; AXIN1KO; AXIN2KO
CSNK1A1KO; AXIN2KO
GSK3AKO; GSK3BKO
CSNK1A1KO; GSK3AKO; GSK3BKO
CSNK1A1KO; GSK3AKO
CSNK1A1KO; GSK3BKO
WT
CSNK1A1KO; AXIN1KO
M (kDa)
HUWE1b
APCa
*
CTNNB1b
AXIN1a
AXIN2a
GSK3Aa
GSK3Ba
CSNK1A1b
GAPDHa
GAPDHb
> 250
> 250
100
75
100
100
75
50
37
37
37
37
B
WT
GSK3AKO
GSK3BKO
GSK3AKO; GSK3BKO
M (kDa)
GSK3A
GSK3B
Na+/K+ ATPase
50
37
100
75
C
- WNT3A
+ WNT3A
Relative WNT reporter
2000
1500
1000
500
0
1
163
1
152
1
131
1303
1457
WT
GSK3AKO
GSK3BKO
GSK3AKO; GSK3BKO
D
Relative HUWE1
150
100
50
0
100
78
100
90
89
89
68
93
120
101
96
75
WT
CSNK1A1KO
APCKO
APCKO; CSNK1A1KO
AXIN1KO; AXIN2KO
CSNK1A1KO; AXIN1KO; AXIN2KO
CSNK1A1KO; AXIN1KO
CSNK1A1KO; AXIN2KO
GSK3AKO; GSK3BKO
CSNK1A1KO; GSK3AKO; GSK3BKO
CSNK1A1KO; GSK3AKO
CSNK1A1KO; GSK3BKO

S5 Fig

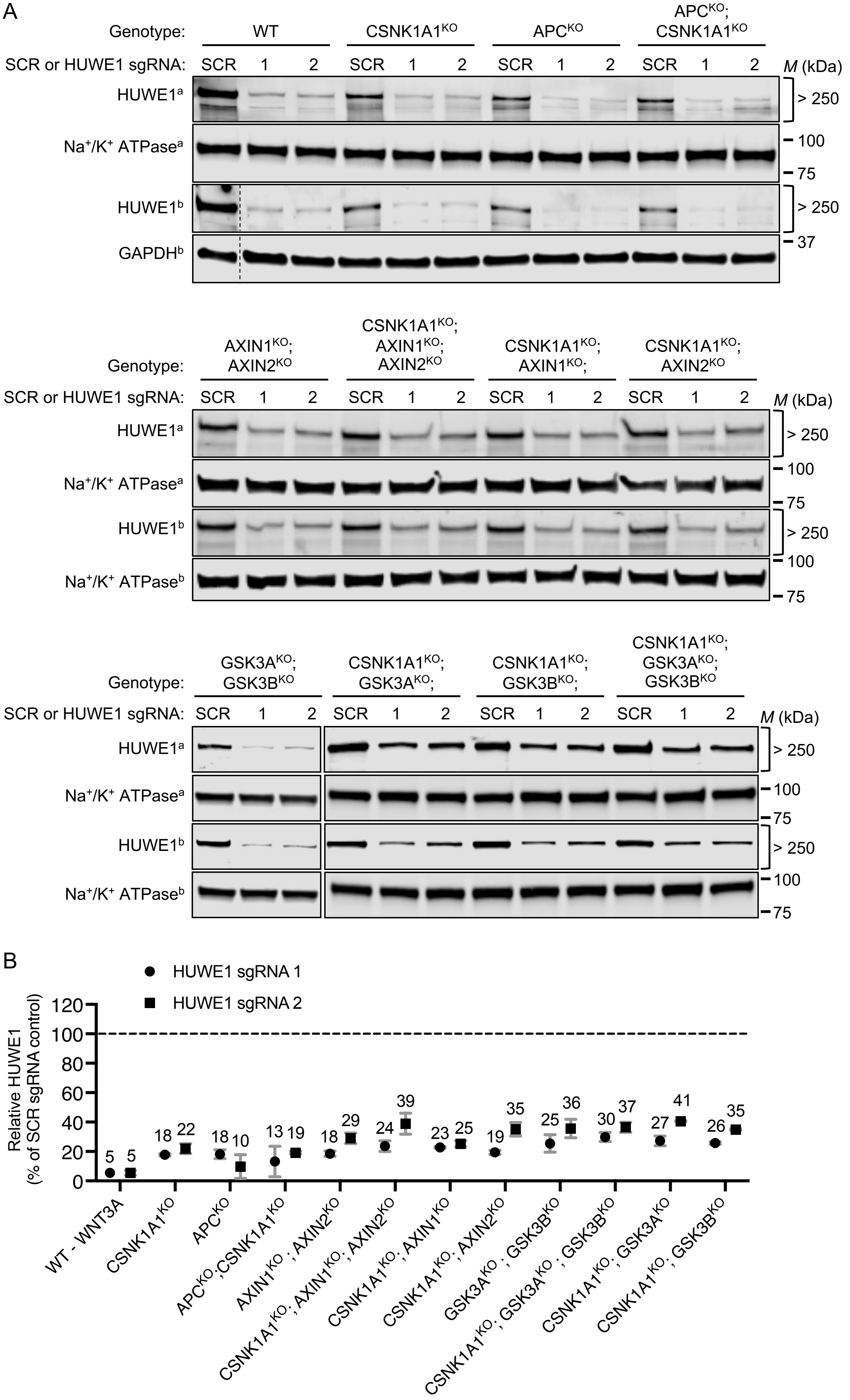
A
Genotype:
WT
CSNK1A1KO
APCKO
APCKO; CSNK1A1KO
SCR or HUWE1 sgRNA:
SCR 1 2
M (kDa)
HUWE1a
Na+/K+ ATPasea
HUWE1b
GAPDHb
> 250
100
75
37
AXIN1KO; AXIN2KO
CSNK1A1KO; AXIN1KO; AXIN2KO
CSNK1A1KO; AXIN1KO;
CSNK1A1KO; AXIN2KO
Na+/K+ ATPaseb
GSK3AKO; GSK3BKO
CSNK1A1KO; GSK3AKO;
CSNK1A1KO; GSK3BKO;
CSNK1A1KO; GSK3AKO; GSK3BKO
B
HUWE1 sgRNA 1
HUWE1 sgRNA 2
Relative HUWE1 (% of SCR sgRNA control)
0
20
40
60
80
100
120
5 5
18 22
18 10
13 19
18 29
24 39
23 25
19 35
25 36
30 37
27 41
26 35
WT - WNT3A
CSNK1A1KO
APCKO
APCKO;CSNK1A1KO
AXIN1KO ; AXIN2KO
CSNK1A1KO; AXIN1KO; AXIN2KO
CSNK1A1KO; AXIN1KO
CSNK1A1KO; AXIN2KO
GSK3AKO ; GSK3BKO
CSNK1A1KO; GSK3AKO ; GSK3BKO
CSNK1A1KO; GSK3AKO
CSNK1A1KO; GSK3BKO

S6 Fig

A HEK293T-7TG

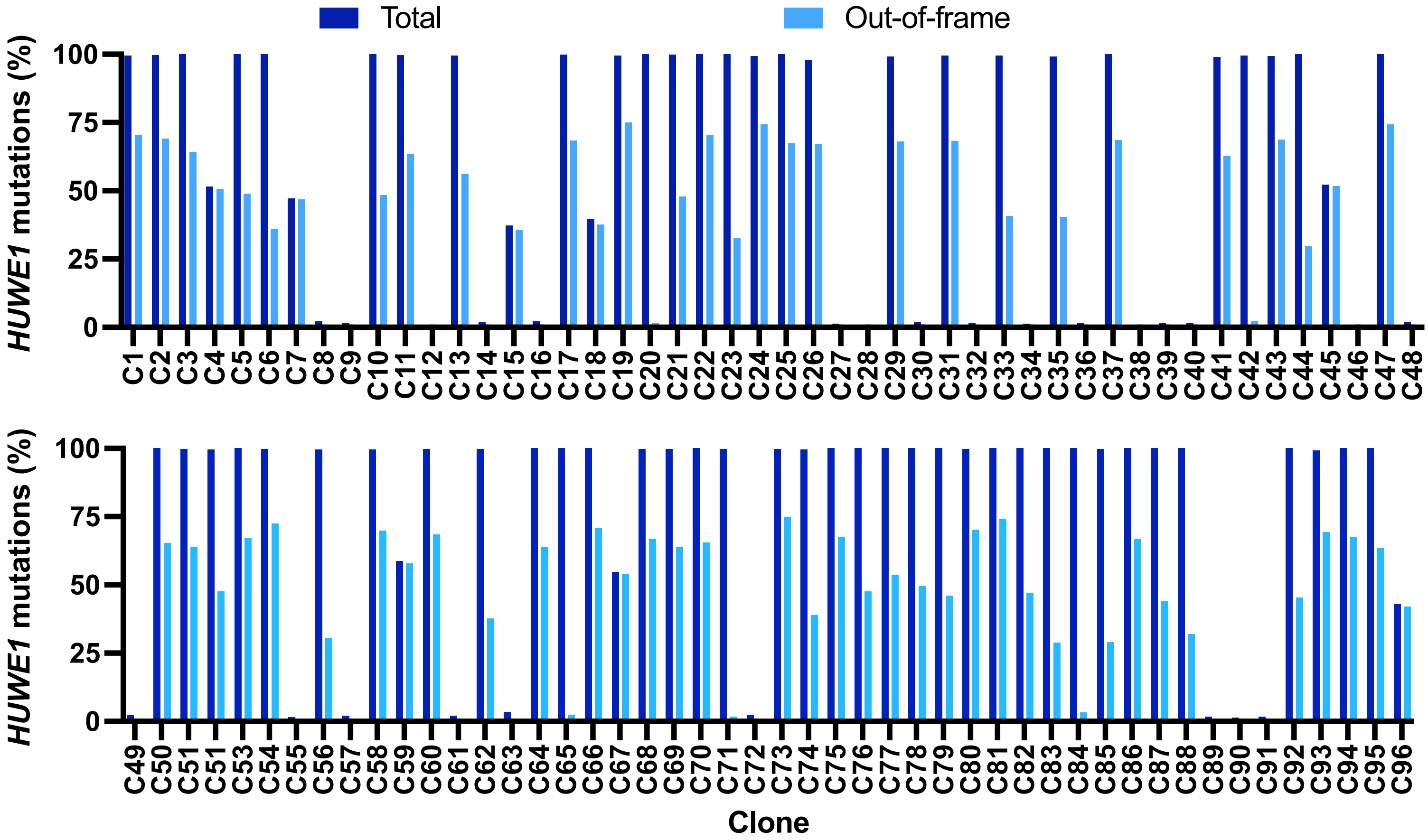

B HEK293T-7TG CSNK1A1[KO]

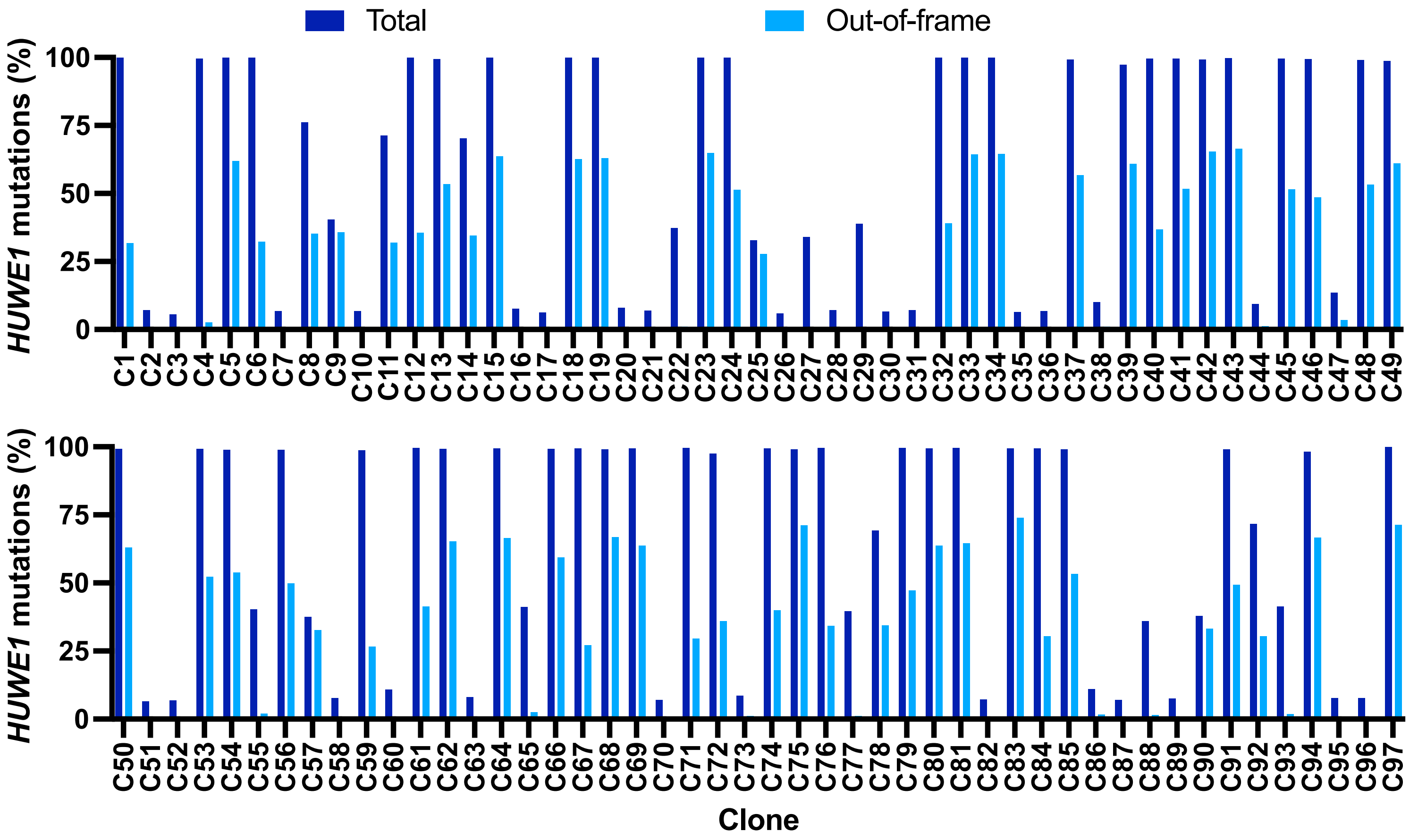

Supplement: Supplement 3 [file NIHPP2024.02.02.578552v2-supplement-3.pdf]
