# Supplementary material for: The ubiquitin ligase HUWE1 enhances WNT signaling by antagonizing destruction complex-mediated β-catenin degradation and through a mechanism independent of β-catenin stability
Source: bioRxiv. 2024 Feb 2:2024.02.02.578552. Preprint. [Version 1] doi: 10.1101/2024.02.02.578552 (PMC10896346; doi:10.1101/2024.02.02.578552)
Supplement: Supplementary file 1 [file NIHPP2024.02.02.578552v1-supplement-3.pdf]

bioRxiv preprint doi: https://doi.org/10.1101/2024.02.02.578552; this version posted February 2, 2024. The copyright holder for this preprint (which was not certified by peer review) is the author/funder. This article is a US Government work. It is not subject to copyright under 17 USC 105 and is also made available for use under a CC0 license.

S1 Fig. HUWE1 and AXIN1 reciprocally regulate WNT signaling by modulating GSK3A/GSK3B-dependent CTNNB1 phosphorylation and abundance.
(A, B) Immunoblots of soluble CTNNB1 (A) and non-phospho-CTNNB1 (S33/S37/T41) (B) used for quantification in Fig 1B and 1C, respectively. (C) Immunoblot analysis of the indicated cell lines used in A, B, and in Fig 1. The polyclonal cell populations overexpressing AXIN1 were generated as described in Materials and methods. AXIN1 abundance (AXIN1 intensity normalized to GAPDH intensity), relative to untreated CSNK1A1^{KO} cells, is shown below the blots.

S2 Fig. HUWE1 enhances WNT signaling through a mechanism independent of CTNNB1 stability.
(A) Genomic nucleotide and corresponding amino acid sequences comprising the CTNNB1 phosphodegron of WT HAP1-7TGP and CTNNB1$^{ST\text{-}A}$ cells. The kinases that phosphorylate S or T residues in the phosphodegron are indicated. Nucleotides and amino acids in red represent mutations. (B) Immunoblots of soluble HUWE1 and CTNNB1 for the indicated cell lines. The CTNNB1 immunoblots were used for quantification in Fig 2A. (C-E) Treatment of CTNNB1$^{ST\text{-}A}$ cells with WNT3A does not promote further accumulation of soluble CTNNB1 and does not further increase WNT target gene expression. Cells were treated with 50% WNT3A CM for 24 hr where indicated. (C) Immunoblots of total CTNNB1 used for quantification in D. (D) Total

bioRxiv preprint doi: https://doi.org/10.1101/2024.02.02.578552; this version posted February 2, 2024. The copyright holder for this preprint (which was not certified by peer review) is the author/funder. This article is a US Government work. It is not subject to copyright under 17 USC 105 and is also made available for use under a CC0 license.

CTNNB1 abundance (CTNNB1 intensity normalized to GAPDH intensity, average ± SD from duplicate immunoblots), reported as percentage of the CTNNB1abundance in untreated CTNNB1$^{ST\text{-}A}$ cells. Significance was determined by unpaired t-test with Welch's correction. (E) mRNA abundance (average ± SD *AXIN2*, *RNF43*, *TNFRSF19*, or *NKD1* mRNA normalized to *HPRT1* mRNA, each measured in triplicate reactions) reported as the percentage of the mRNA abundance in untreated CTNNB1$^{ST\text{-}A}$ cells. (F) WNT reporter activity (median EGFP fluorescence from 5,000 singlets) for the indicated cell lines, relative to the average for CTNNB1$^{ST\text{-}A}$ cells. Each circle represents a unique clonal cell line, and the average of 9-12 independent clones for each genotype is indicated by a horizontal line and quantified above each group of circles. Significance was determined by unpaired t-test with Welch's correction.

S4 Fig. HUWE1 enhances WNT signaling through mechanisms mediated by a subset of DC components including APC, AXIN1 and GSK3A or GSK3B.
(A) Immunoblot analysis of total protein from the indicated clonal cell lines used for CRISPRi-mediated HUWE1 KD. * indicates a non-specific band observed with the anti-APC antibody (Sigma). The "a" and "b" superscripts next to the protein names indicate which of two membranes the corresponding strips were cut from. Dashed vertical lines indicate a

bioRxiv preprint doi: https://doi.org/10.1101/2024.02.02.578552; this version posted February 2, 2024. The copyright holder for this preprint (which was not certified by peer review) is the author/funder. This article is a US Government work. It is not subject to copyright under 17 USC 105 and is also made available for use under a CC0 license.

rearrangement of samples within the same blot. (B, C) GSK3A and GSK3B are functionally redundant in WNT signaling in HAP1 cells. (B) Immunoblot analysis of GSK3A and/or GSK3B KO cell lines. (C) WNT reporter activity (median EGFP fluorescence from 50,000 singlets was measured for biological duplicates of a single clone, and the average ± SD of the two measurements was calculated) relative to untreated WT HAP1-7TGP cells. The same cell lines were used in B and C. (D) Total HUWE1 abundance (average ± SD from triplicate HUWE1 dot blots normalized to total protein) relative to WT HAP1-7TGP cells, quantified from dot blots of the indicated clonal cell lines used for CRISPRi-mediated HUWE1 KD. Significance was determined by unpaired t-test with Welch's correction and the difference in HUWE1 abundance between each mutant cell line and WT HAP1-7TGP cells was not significant (not depicted in the graph).

S5 Fig. Quantification of CRISPRi-mediated HUWE1 KD in various genetic backgrounds. (A) Immunoblots of total HUWE1 used for quantification in B. Dashed vertical lines indicate a rearrangement of samples within the same blot. The superscripts "a" and "b" next to the protein names indicate replicates. (B) Analysis of HUWE1 KD efficacy in each genotype. Two polyclonal cell populations targeted with HUWE1 sgRNAs (1 and 2) and one polyclonal cell population targeted with SCR sgRNA were derived for each genotype as described in Materials and methods. HUWE1 KD efficacy was calculated as HUWE1 abundance (average HUWE1 intensity normalized to either Na^+/K^+ ATPase or GAPDH intensity from duplicate immunoblots) in the cell populations targeted with each HUWE1 sgRNA relative the cell populations targeted with the SCR sgRNA control.

bioRxiv preprint doi: https://doi.org/10.1101/2024.02.02.578552; this version posted February 2, 2024. The copyright holder for this preprint (which was not certified by peer review) is the author/funder. This article is a US Government work. It is not subject to copyright under 17 USC 105 and is also made available for use under a CC0 license.

Supporting Information:

S1 File. List of clonal cell lines used in this study. Clones in which a single gene was targeted using CRISPR/Cas9, and double- or triple-mutant clones in which multiple genes were disrupted using CRISPR/Cas9 are described in two separate spreadsheets labeled accordingly. For cell lines engineered using CRISPR/Cas9, when more than one clone was generated using the same CRISPR guide, the 'Clone Name' column indicates the generic name used throughout the manuscript to describe the genotype, and the 'Clone #' column identifies the specific allele in each individual clone. The 'CRISPR guide' column indicates the name of the guide used, which is the same as that of the oligos encoding sgRNAs (see Materials and methods, and S2 File). The 'Genomic Sequence' column shows 80 bases of genomic sequence (5' relative to the gene is to the left) surrounding the target site. For each group of clones made using the same CRISPR guide (separated by gray spacers), the 'Genomic Sequence' column is headlined by the reference WT genomic sequence (obtained from RefSeq), with the guide sequence colored blue. The site of the double strand cut made by Cas9 is between the two underlined bases. Sequencing results for individual clones are indicated below the reference sequence. Some WT clones are indicated as such and were used as controls. For mutant clones, mutated bases are colored red (dashes represent deleted bases, three dots are used to indicate that a deletion continues beyond the 80 bases of sequence shown, and large insertions are indicated in brackets), and the nature of the mutation, the resulting genotype and any pertinent observations are also described. The figures in which each clone was used are also indicated. For double- or triple- mutant clones, the CRISPR guide used, the genomic sequence, the mutation, the genotype and any observations pertaining to each of the two or three targeted genes are designated '1', '2' and '3' in the column headings, and are shown under green, orange and purple spacers, respectively.

S2 File. List of oligonucleotides and primers used for generation and characterization of clonal cell lines engineered using CRISPR/Cas9 nuclease (CRISPRn), base editing, and CRISPRi are described in three separate spreadsheets labeled accordingly. The names and sequences of pairs of oligonucleotides encoding sgRNAs (which were cloned into the respective vectors for each application as described in Materials and methods) are shown in columns A and B, respectively. Additionally, for CRISPRn and base editing, the names and sequences of pairs of primers used to amplify corresponding genomic regions flanking sgRNA target sites are shown in columns C and D, respectively, and the names and sequences of single primers used to sequence the amplified target sites are shown in columns E and F, respectively.

S1 Fig

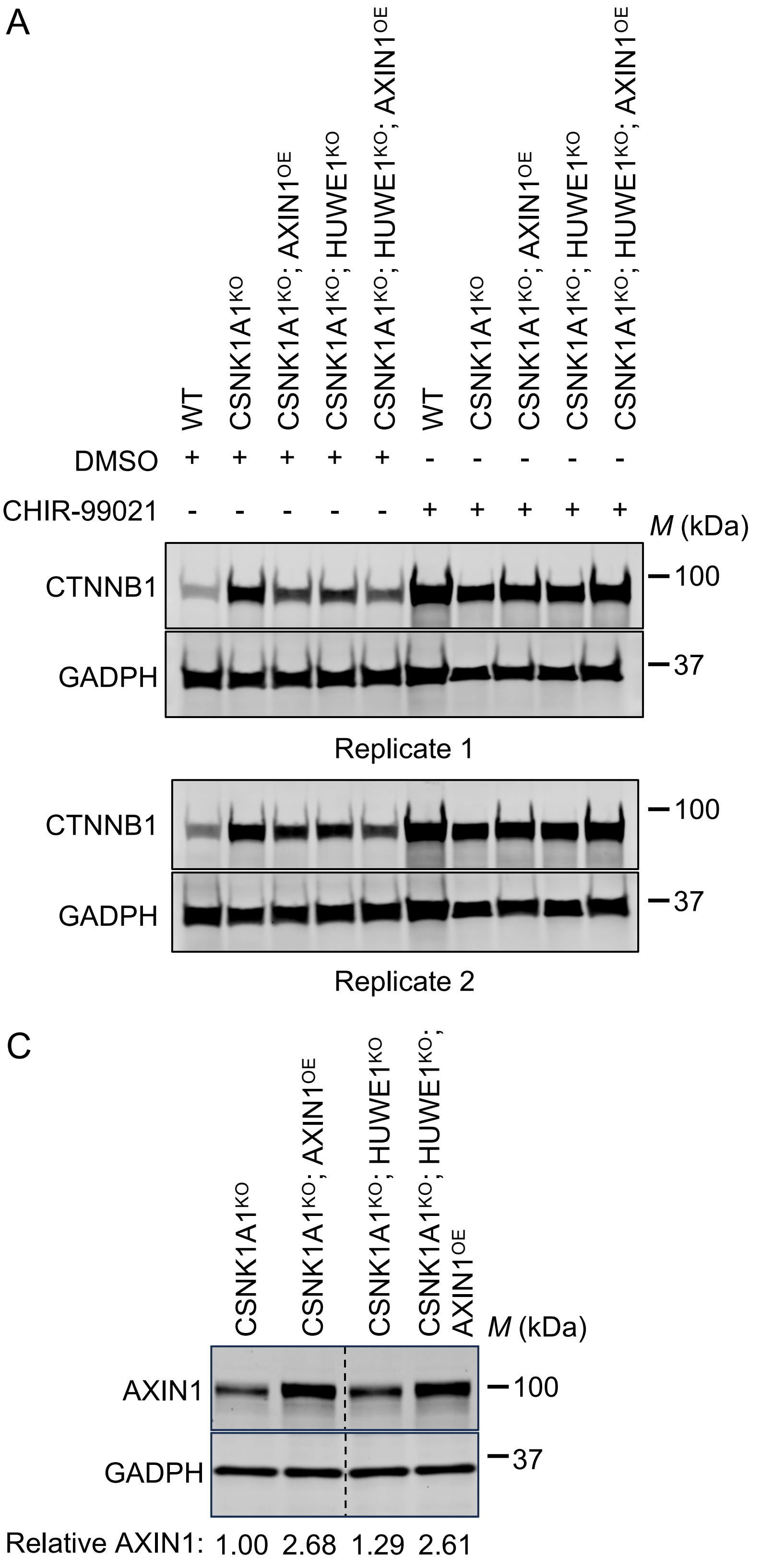
A
WT
CSNK1A1KO
CSNK1A1KO; AXIN1OE
CSNK1A1KO; HUWE1KO
CSNK1A1KO; HUWE1KO; AXIN1OE
DMSO
CHIR-99021
M (kDa)
CTNNB1
GADPH
100
37
Replicate 1
Replicate 2
B
Non-phospho-CTNNB1
Replicate 1
Replicate 2
C
AXIN1
Relative AXIN1: 1.00 2.68 1.29 2.61

S2 Fig

A

CTNNB1 phosphodegron

```
CTNNB1WT:   CTG GAC TCT GGA ATC CAT TCT GGT GCC ACT ACC ACA GCT CCT TCT CTG
CTNNB1ST-A: CTG GAC TCT GGA ATC CAT GCT GGT GCC ACT GCC ACA GCT CCT GCT CTG

CTNNB1WT  : L31,D32,S33,G34,I35,H36,S37,G38,A39,T40,T41,T42,A43,F44,S45,L36
CTNNB1ST-A : L31,D32,S33,G34,I35,H36,A37,G38,A39,T40,A41,T42,A43,F44,A45,L36

Kinase:        GSK3A/B        GSK3A/B        GSK3A/B        CSNK1A1
```

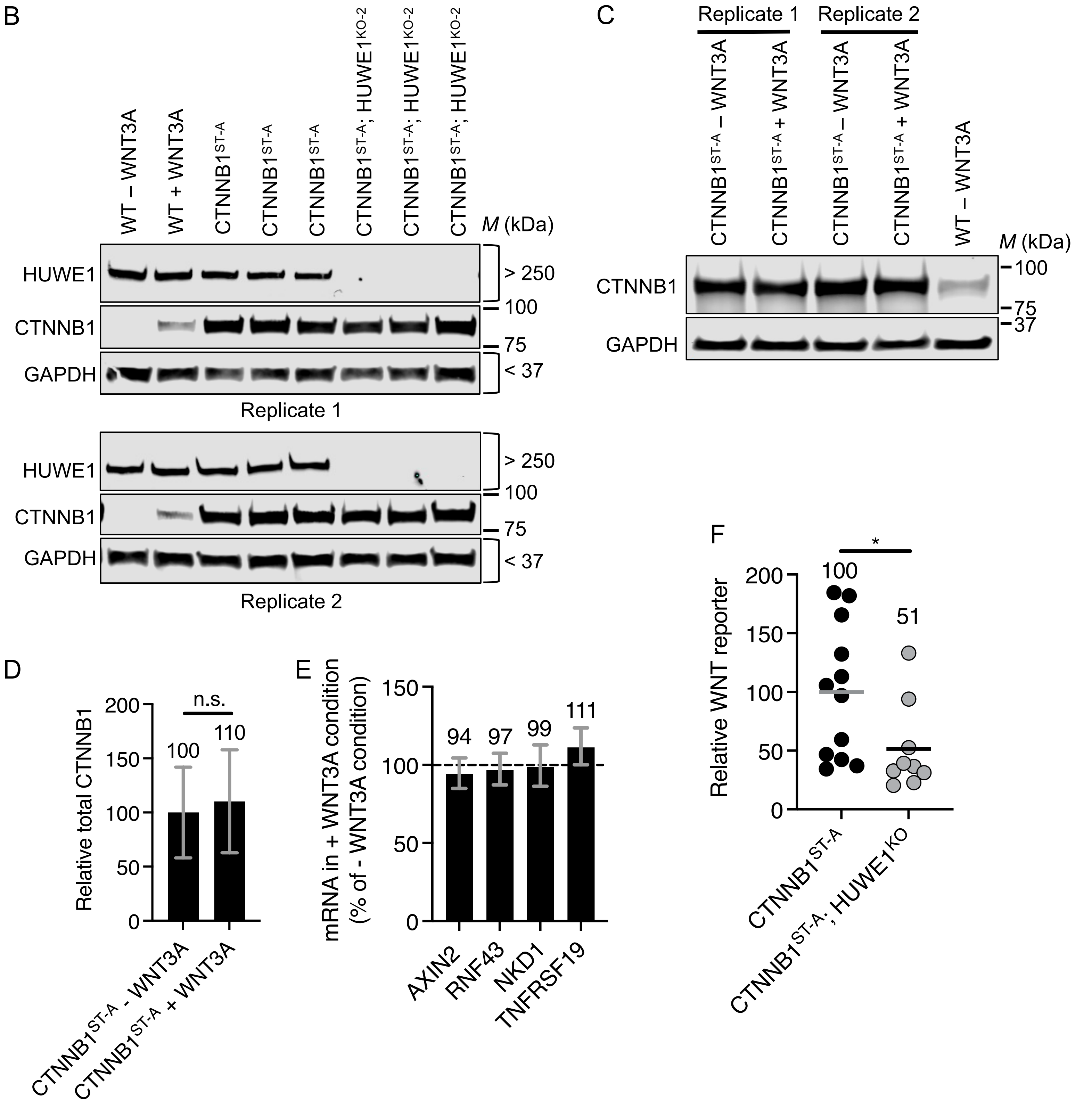

S4 Fig

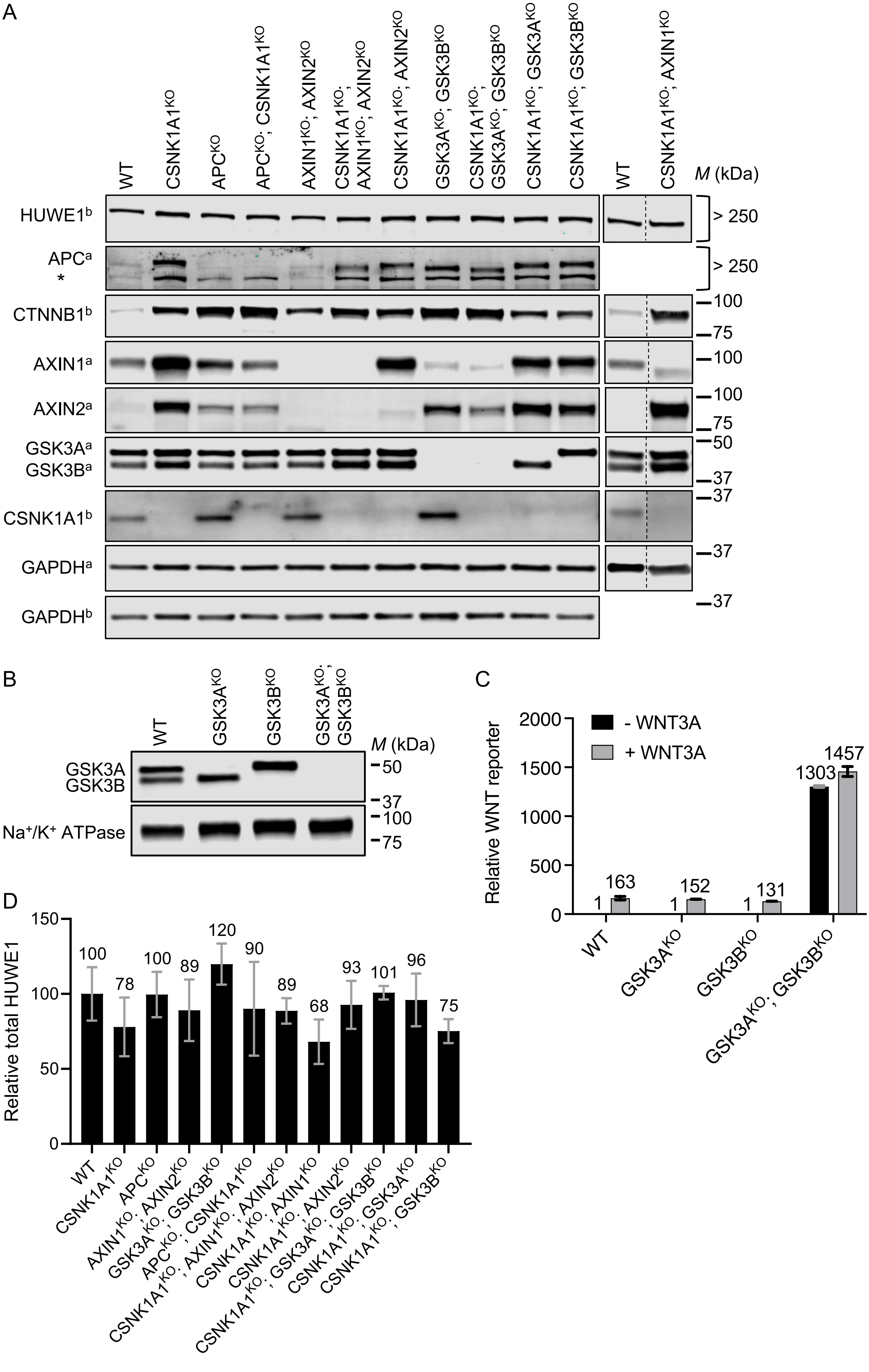
A
WT
CSNK1A1KO
APCKO
APCKO; CSNK1A1KO
AXIN1KO; AXIN2KO
CSNK1A1KO; AXIN1KO; AXIN2KO
CSNK1A1KO; AXIN2KO
GSK3AKO; GSK3BKO
CSNK1A1KO; GSK3AKO; GSK3BKO
CSNK1A1KO; GSK3AKO
CSNK1A1KO; GSK3BKO
WT
CSNK1A1KO; AXIN1KO
M (kDa)
HUWE1b
APCa
*
CTNNB1b
AXIN1a
AXIN2a
GSK3Aa
GSK3Ba
CSNK1A1b
GAPDHa
GAPDHb
> 250
> 250
100
75
100
100
75
50
37
37
37
37
B
WT
GSK3AKO
GSK3BKO
GSK3AKO; GSK3BKO
M (kDa)
GSK3A
GSK3B
Na+/K+ ATPase
50
37
100
75
C
- WNT3A
+ WNT3A
Relative WNT reporter
2000
1500
1000
500
0
1
163
1
152
1
131
1303
1457
WT
GSK3AKO
GSK3BKO
GSK3AKO; GSK3BKO
D
Relative total HUWE1
150
100
50
0
100
78
100
89
120
90
89
68
93
101
96
75
WT
CSNK1A1KO
APCKO
AXIN1KO; AXIN2KO
GSK3AKO; GSK3BKO
APCKO; CSNK1A1KO
CSNK1A1KO; AXIN1KO; AXIN2KO
CSNK1A1KO; AXIN1KO
CSNK1A1KO; AXIN2KO
CSNK1A1KO; GSK3AKO; GSK3BKO
CSNK1A1KO; GSK3AKO
CSNK1A1KO; GSK3BKO

S5 Fig

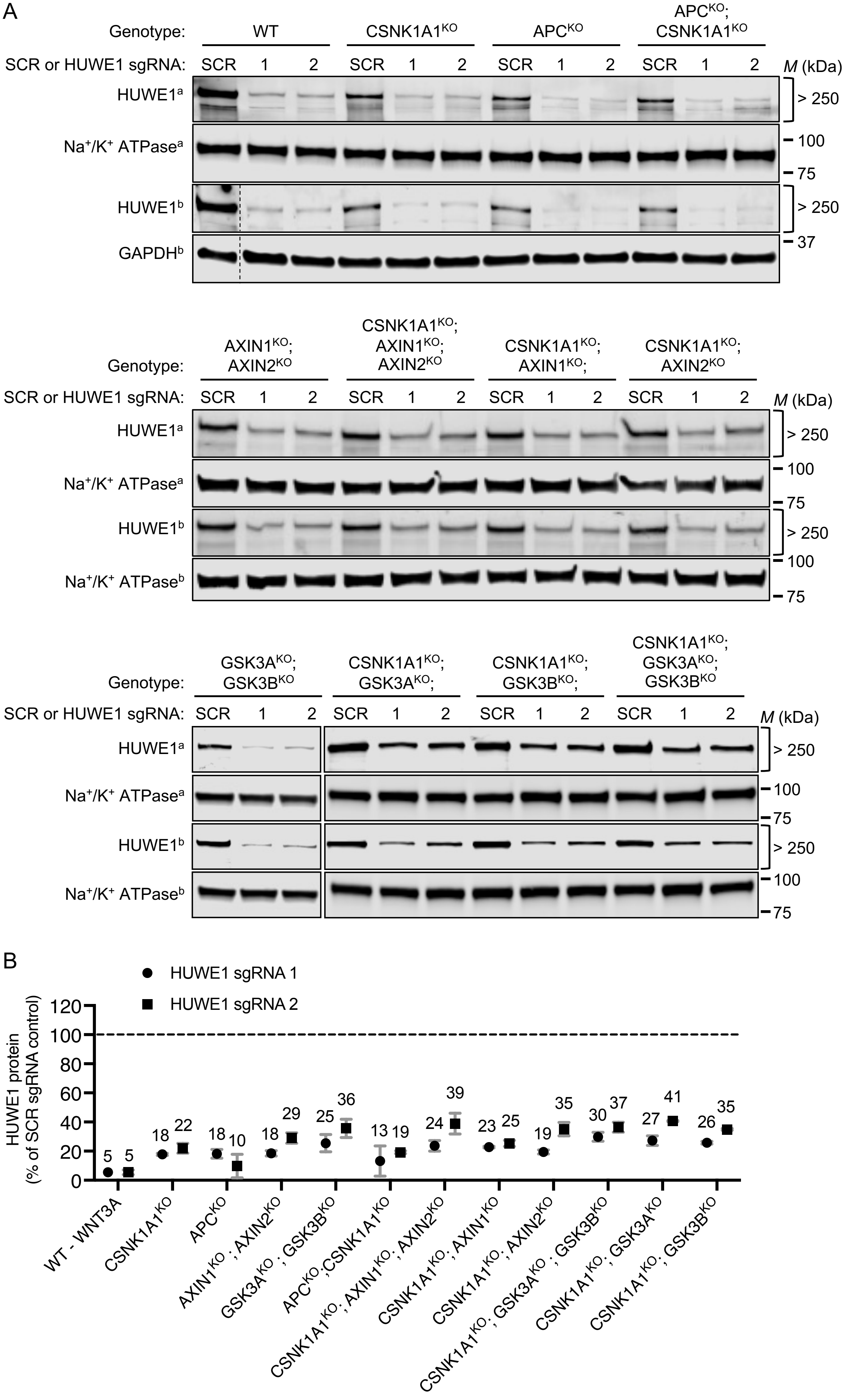
A
Genotype:
WT
CSNK1A1KO
APCKO
APCKO; CSNK1A1KO
SCR or HUWE1 sgRNA:
SCR 1 2
M (kDa)
HUWE1a
Na+/K+ ATPasea
HUWE1b
GAPDHb
> 250
100
75
37
AXIN1KO; AXIN2KO
CSNK1A1KO; AXIN1KO; AXIN2KO
CSNK1A1KO; AXIN1KO;
CSNK1A1KO; AXIN2KO
Na+/K+ ATPaseb
GSK3AKO; GSK3BKO
CSNK1A1KO; GSK3AKO;
CSNK1A1KO; GSK3BKO;
CSNK1A1KO; GSK3AKO; GSK3BKO
B
HUWE1 sgRNA 1
HUWE1 sgRNA 2
HUWE1 protein (% of SCR sgRNA control)
120
100
80
60
40
20
0
5 5
18 22
18 10
18 29
25 36
13 19
24 39
23 25
19 35
30 37
27 41
26 35
WT - WNT3A
CSNK1A1KO
APCKO
AXIN1KO ; AXIN2KO
GSK3AKO ; GSK3BKO
APCKO;CSNK1A1KO
CSNK1A1KO; AXIN1KO; AXIN2KO
CSNK1A1KO; AXIN1KO
CSNK1A1KO; AXIN2KO
CSNK1A1KO; GSK3AKO ; GSK3BKO
CSNK1A1KO; GSK3AKO
CSNK1A1KO; GSK3BKO
